# Screen Printing of Surface-Modified Barium Titanate/Polyvinylidene Fluoride Nanocomposites for High-Performance Flexible Piezoelectric Nanogenerators

**DOI:** 10.3390/nano12172910

**Published:** 2022-08-24

**Authors:** Hai Li, Sooman Lim

**Affiliations:** Department of Flexible and Printable Electronics, LANL-JBNU Engineering Institute, Jeonbuk National University, Jeonju 54896, Korea

**Keywords:** screen printing, surface modification, piezoelectric nanogenerators, sensors

## Abstract

Piezoelectric energy harvesters are appealing for the improvement of wearable electronics, owing to their excellent mechanical and electrical properties. Herein, screen-printed piezoelectric nanogenerators (PENGs) are developed from triethoxy(octyl)silane-coated barium titanate/polyvinylidene fluoride (TOS-BTO/PVDF) nanocomposites with excellent performance based on the important link between material, structure, and performance. In order to minimize the effect of nanofiller agglomeration, TOS-coated BTO nanoparticles are anchored onto PVDF. Thus, composites with well-distributed TOS-BTO nanoparticles exhibit fewer defects, resulting in reduced charge annihilation during charge transfer from inorganic nanoparticles to the polymer. Consequently, the screen-printed TOS-BTO/PVDF PENG exhibits a significantly enhanced output voltage of 20 V, even after 7500 cycles, and a higher power density of 15.6 μW cm^−2^, which is 200 and 150% higher than those of pristine BTO/PVDF PENGs, respectively. The increased performance of TOS-BTO/PVDF PENGs is due to the enhanced compatibility between nanofillers and polymers and the resulting improvement in dielectric response. Furthermore, as-printed devices could actively adapt to human movements and displayed excellent detection capability. The screen-printed process offers excellent potential for developing flexible and high-performance piezoelectric devices in a cost-effective and sustainable way.

## 1. Introduction

Printing technology has rapidly advanced owing to the increasing demand for printed electronics, which use analog or digital printing technologies to print electrical circuits or devices [1,2,3,4]. The development of printed electronics on flexible substrates has the potential to fundamentally change the way of manufacturing electrical devices. Emerging electronic technologies involving new material synthesis, unique device concepts, new functionality, and new manufacturing processes have revolutionized the microelectronics sector, which traditionally focused on silicon and microfabrication techniques [5,6,7,8,9]. Owing to the increase in the number of printing-based electronic devices, several semiconductors, and conductive and dielectric materials have been produced as inks for various printing processes [10,11,12]. Electroactive polymers are among the most promising materials for flexible electronics, and piezoelectric materials are among those that can be printed with promising outcomes [13,14]. Electrical signals generated by piezoelectric materials in response to mechanical stress, vibration, or deformation are receiving increasing research interest [15,16]. This lays the groundwork for its use in wearable electronics, such as sensors, actuators, and energy harvesters [17,18,19].

Polyvinylidene fluoride (PVDF) and its copolymers are the most suitable piezoelectric materials for printed sensors and actuators because of their biocompatibility, toughness, lightness, and mechanical adaptability [20,21,22]. The viscosity and density of PVDF inks can be tailored to match the printing equipment by solubilizing them in acetone or *N*,*N*-dimethylformamide (DMF). Furthermore, PVDF can be printed on a variety of soft substrates, including polyethylene terephthalate (PET) or paper, which are most commonly utilized in sensors [23,24]. In particular, PVDF has five crystal forms: α-, β-, γ-, δ-, and ε-phases [25,26,27]. The α-phase is nonpolar, whereas the γ- and δ-phases are weakly polar. Owing to its high polarity, the β-phase exhibits optimal piezoelectric and ferroelectric properties. The β-phase can be obtained via applying a high pressure/electric field [28,29], mechanical stretching [30], electrospinning [31], or adding nucleating fillers [32,33]. However, despite the high β-phase concentrations of PVDF and its copolymers, their low piezoelectric coefficients (d_33_ values) prevent their application in piezoelectric sensors for wearable electronics. Therefore, inorganic materials, such as lead zirconium titanate (PZT) and barium titanate (BTO), have been employed to fabricate high-performance piezoelectric composites [34,35,36,37]. However, inorganic nanoparticles are unsuitable for fabricating flexible and high-performance piezoelectric nanogenerators (PENGs) because of their easy agglomeration due to their large surface area and high surface activity [38,39]. In ceramic powders, triethoxy(octyl)silane (TOS) has been extensively utilized as a dispersant for nanoparticle suspensions to increase dispersion. Furthermore, TOS is a low-cost, non-toxic silane with concentrated reactive groups and strong chelating activity [40,41]. Therefore, the TOS modification of BTO nanoparticles may be a promising strategy to improve the piezoelectric performance of ceramic–polymer nanocomposites, facilitate uniform nanoparticle dispersion in the polymer matrix and enhance interfacial compatibility. However, to the best of our knowledge, TOS-modified BTO has not been used to alter the nanoparticle interface or enhance the output performance of PENGs.

In this paper, we present a novel screen-printing method for fabricating flexible piezoelectric sensors and energy harvesters using a high-performance TOS-BTO/PVDF composite and silver ink. As all of the inks are non-toxic and use environmentally friendly solvents, the technique promotes long-term large-area sensor technology. To print the ceramic–polymer nanocomposite using a screen printer, we synthesized TOS-modified BTO nanoparticles and distributed them into the PVDF polymer matrix to form a nanocomposite with an appropriate viscosity coefficient. The TOS surface modification agent-induced strong hydrogen bonds with PVDF to form a high β-phase content, thereby resulting in a pronounced performance of the nanocomposites. Therefore, the TOS-BTO/PVDF PENG exhibited a noticeable increase in voltage (20 V) and power density (15.6 μW cm^−2^), which were 200 and 150% higher than those of pristine BTO/PVDF PENGs, respectively. Furthermore, wearable electronics, small-sized energy harvesters, and soft robotics may benefit from the wide variety of functionalities and excellent electromechanical coupling properties of TOS-BTO/PVDF PENGs.

## 2. Materials and Methods

### 2.1. Preparation of TOS-BTO

First, acetic acid (Daejung Inc., Busan, Korea) was used to adjust the pH of an ethanol–water mixed solution (95:5 (*v*/*v*), Daejung Inc.) to 3–5. Thereafter, TOS (1 mL, Sigma-Aldrich, St. Louis, MO, USA) was added to the combination solution and hydrolyzed for 1 h at 25 °C. Subsequently, BTO nanoparticles (0.5 g, average diameter: 100 nm, US Research Nanomaterials Inc., Houston, TX, USA) were added, and the mixture was manually agitated for 3 h at 60 °C to enable TOS to adhere to the BTO surface. Thereafter, the mixture was centrifuged for 10 min at 8000 rpm to remove any unreacted silane reagent and rinsed several times with ethanol. Finally, the collected TOS-BTO nanoparticles were placed in a vacuum oven at 60 °C for 24 h to eliminate any residual solvent.

### 2.2. Screen Printing of PENGs

**Figure 1:**
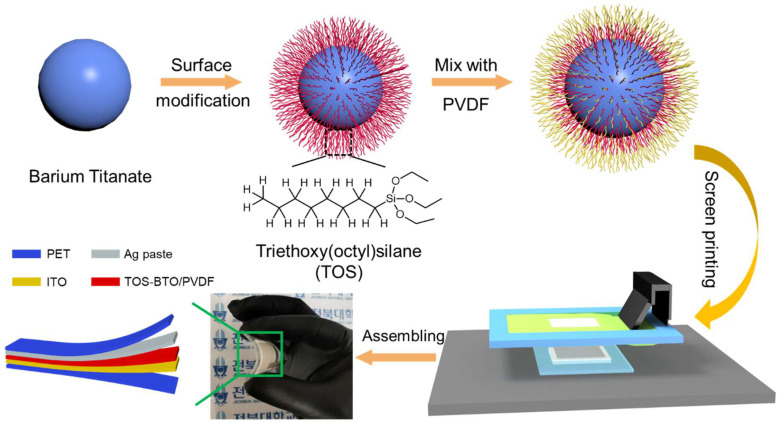
Schematic of the fabrication of the screen-printed TOS-BTO/PVDF piezoelectric sensor.

A satisfactory dispersion solution of TOS-BTO nanoparticles (0.1 g) in DMF solution (10 mL, Daejung Inc.) was obtained to prepare screen printing ink. After heating the suspension solutions to 30 °C, PVDF polymer (1 g, Mw-530 000, Sigma–Aldrich) was added, and the mixture was mechanically stirred for 12 h. The screen-printing process was performed using a screen printer (AMX-1242T Semi-Auto screen printer, Bucheon, Korea) with an emulsion screen mesh (400 mesh count, thread per inch, 830 μm opening, 39 µm mesh thickness), as shown in Figure 1. A squeegee was used to force ink transfer through the open mesh of the desired mask design onto the substrate. The squeegee was used to drive the ink into the substrate (indium tin oxide-coated PET (ITO-PET)) via the open mesh of the desired mask design at a 45° angle with the mesh at a speed of 60 mm s^−1^. A printed piezoelectric layer with a thickness of 20 μm was obtained after drying in a vacuum oven at 80 °C for 4 h. Subsequently, a commercial silver paste was printed onto the piezoelectric layer to form surface electrodes using the same process. Finally, the printed piezoelectric samples were poled under a 50 V μm^−1^ electric field at 110 °C for 4 h. For comparison, PVDF films containing untreated BTO were prepared in the same manner.

### 2.3. Measurement and Characterization

The crystalline structures of the as-printed samples were determined using X-ray diffraction patterns with Cu K-alpha radiation (D8 Advance diffractometer, Bruker, Germany). The chemical structure of the printed samples was investigated using Fourier-transform infrared (FTIR) spectroscopy (Bomen MB 100, Bomen, Canada) in the wavenumber range of 400–4000 cm^−1^. Field emission scanning electron microscopy (FESEM, SUPRA 40VP, Carl Zeiss, Germany) was used to analyze the morphologies of the printed samples at the Jeonbuk National University Center for University-wide Research Facilities (CURF, Jeonju, Korea). A Digital Rotational Brookfield Viscometer (Model DVNXRVCJG, Brookfield Engineering Laboratories, Middleboro, MA, USA) was used to measure the rheological properties of the as-prepared inks with shear rates of 0.1–1000 s^−1^. The piezoelectricity (d_33_) of the screen-printed composite film was measured using a quasistatic d_33_ meter (YE2730A, Sinocera, Yangzhou, China). An oscilloscope (KEYSIGHT DSOX2012A, Santa Rosa, CA, USA) and Keithley 2450 source meter were used to test the output voltages and currents of the screen-printed PENGs, respectively.

## 3. Results and Discussion

**Figure 2:**
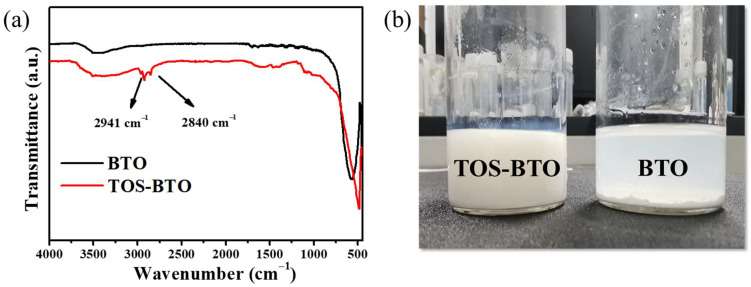
(**a**) FTIR spectra of the BTO and TOS-BTO nanoparticles (inset: chemical structure of the TOS). (**b**) Optical photograph of the TOS-BTO/PVDF and BTO/PVDF inks after 10 d of standing.

In order to verify the existence of functional groups in their surface structures, the FTIR spectra of the TOS-BTO nanoparticles were obtained using an FTIR spectrometer with a measurement range of 4000–500 cm^−1^. FTIR spectra of the pristine BTO nanoparticles were also obtained using the same procedure for comparison. The FTIR spectra of BTO and TOS-BTO nanoparticles are shown in Figure 2a. For the pristine BTO nanoparticles, a distinctive Ti–O vibration peak was located at 529 cm^−1^. In addition, the FTIR spectra did not reveal any further absorption peaks. However, the characteristic peak of the TOS group on the BTO nanoparticles was observed in the FTIR spectra. The absorption peaks that emerged at 2941 and 2840 cm^−1^ were attributed to the stretching vibration of C–H bonds in the form of -CH_2_- and -CH_3_ groups of the TOS-BTO nanoparticles, respectively. Therefore, the presence of these two peaks indicates that nanoparticle surfaces have been grafted with the TOS long carbon chain [40]. Compared to pristine BTO nanoparticles, particle sedimentation was not observed in the polymer matrix solution containing TOS-BTO particles, even after 10 d, as shown in Figure 2b. Therefore, the interaction between the long carbon chain of TOS in the polymer matrix and BTO nanoparticles presumably increases the nanoparticle dispersion and stability in the polymer solution. This is one of the most significant challenges faced by traditional compositing techniques, and it is crucial to ensure the possibility of industrial use with long-term storage.

**Figure 3:**
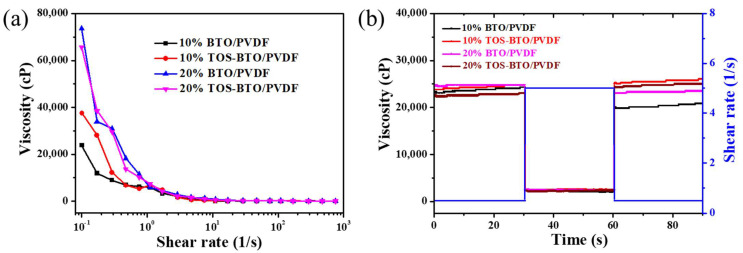
(**a**) Ink viscosity with shear rate and (**b**) thixotropic behavior for PVDF-based ink with various BTO and TOS-BTO nanoparticle contents.

The good printing properties of composite inks with acceptable rheological characteristics may result in thinner lines with smoother edges and be useful in eliminating flaws and bubbles in the printed patterns. A plate–plate rheometer was used to explore the effects of BTO nanoparticle concentration and surface modification characteristics on the rheological behavior of PVDF-based composite inks. Viscosity was determined at various shear rates throughout the steady-state flow step test, as illustrated in Figure 3a. The viscosity of the ink decreased as the shear rate increased, which is characteristic of pseudoplastic fluids. Furthermore, PVDF-based composite inks with higher BTO nanoparticle contents exhibited higher viscosities at the same shear rate owing to the increased degree of aggregation against the dispersion of BTO nanoparticles. In addition, PVDF-based composite inks with surface-modified TOS-BTO nanoparticles displayed lower viscosities at the same shear rate compared to their counterparts with unmodified BTO nanoparticles, owing to the reduced degree of aggregation. The thixotropic properties of the PVDF-based composite inks were examined by retaining the ink at varying shear rates in the three phases, as illustrated in Figure 3b. Viscosity recovery after the initial squeeze stroke was investigated using three separate stages of shear. The first, second, and final stages had shear rates of 0.5, 5, and 0.5 s^−1^, respectively, for 30 s. Viscosity recovery to the first stage, which describes ink elasticity, may impact ink leveling and line creation. A strong hysteresis impact on the viscosity of PVDF-based composite inks was detected when the shear rate increased and decreased, implying that once the shear forces were eliminated, the ink was appropriately leveled in a short time (a few seconds). As a result, the rheological properties of the ink combinations provide effective ink filling and leveling in the empty regions during printing.

**Figure 4:**
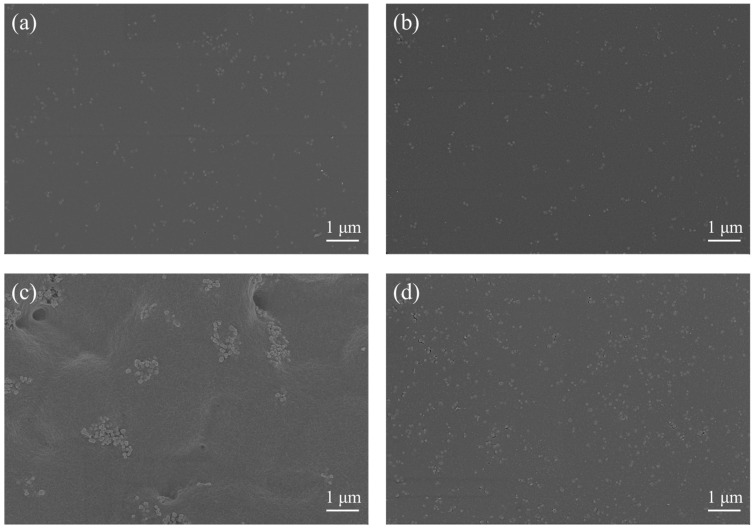
FESEM images of the surfaces of the printed composite films: (**a**) 10% BTO/PVDF, (**b**) 10% TOS-BTO/PVDF, (**c**) 20% BTO/PVDF, and (**d**) 20% TOS-BTO/PVDF.

We then determined that the BTO nanoparticles were distributed in the printed composite films using FESEM. Figure 4a,b show the surface morphology of the nanocomposites with various quantities of TOS-BTO or BTO nanoparticles in the PVDF polymer matrix. The nanoparticles are uniformly and homogeneously distributed in the polymer matrix when 10% BTO and TOS-BTO nanoparticles are employed, as shown in Figure 4a,b. However, when 20 wt% nanoparticles were used, there were considerably more differences in the surface morphology between the BTO/PVDF and TOS-BTO/PVDF composite films. Figure 4d shows that the nanoparticles were uniformly distributed in the PVDF matrix, with no apparent interface hole defects or fractured sections of the material, whereas Figure 4c shows the pristine BTO/PVDF counterpart with many holes and aggregated clusters. The hydrogen bonding of the –CF in the PVDF polymer matrix and the characteristics of –CH on the TOS-BTO nanoparticles may explain these phenomena, which improve the phase compatibility of the nanocomposites during mixing and reduce the contact interface hole defects in ceramic–polymer nanocomposites.

**Figure 5:**
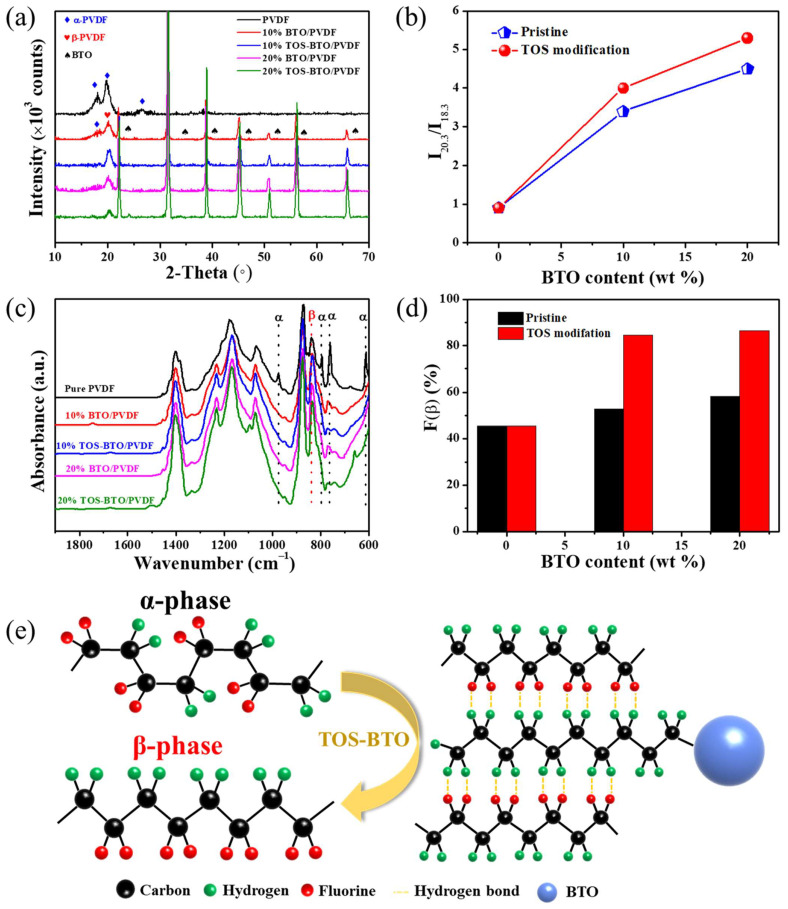
(**a**) XRD spectra of pristine PVDF, BTO/PVDF and TOS-BTO/PVDF film. (**b**) I_20.3_/I_18.3_ ratios of the samples calculated from XRD spectra. (**c**) FTIR spectra of pristine PVDF, BTO/PVDF and TOS-BTO/PVDF film. (**d**) Variation in the F(β) value of the as-prepared samples. (**e**) Schematic of phase transformation between TOS-BTO nanoparticles and the PVDF matrix.

The β-phase content determines the piezoelectricity of PVDF. The crystalline phase composition of the as-prepared samples was investigated using XRD to validate the changes in the phase composition of PVDF. Figure 5a shows the XRD patterns of the as-printed nanocomposites with different BTO nanoparticle weight fractions. The typical characteristic peaks of pure PVDF are at 2θ = 18.3° and 26.5°, which are related to the α-phase of PVDF. The (020) and (021) reflections of PVDF were attributed to these two peaks. In addition, seven distinct diffraction peaks were clearly observed in the XRD pattern, which corresponds to the reflections of the (200), (100), (110), (111), (200), (210), and (211) crystal planes. The appearance of these peaks in the nanocomposite XRD patterns shows that the crystalline characteristics of the TOS-BTO nanoparticles correspond to a ferroelectric tetragonal phase, and the crystal phase of TOS-BTO nanoparticles was not changed by the TOS surface modification. After blending the TOS-BTO nanoparticles with the PVDF polymer, the intensity of the α-phase peak decreased significantly. Accordingly, the diffraction peaks at 20.5°, corresponding to the reflection of the sum of the (110) and (200) crystal planes, confirm the emergence of the crystal β-phase in the TOS-BTO/PVDF nanocomposite, whereas the related peaks of BTO/PVDF are not visible. The findings reveal that the TOS-BTO nanoparticles may operate as nucleation agents in the nanocomposite film, promoting better dipole alignment and the formation of higher β-phase content in the PVDF. Furthermore, by calculating the ratio of the intensities (I_20.3_ and I_18.3_), the β-phase and α-phase contents can be qualitatively estimated, as shown in Figure 5b. The surface-modified BTO nanoparticles significantly influenced the β-phase content. Compared to the pristine BTO/PVDF composite film, which exhibits I_20.3_/I_18.3_ ratios of 3.4 and 4.5 corresponding to 10% and 20% BTO nanoparticles, respectively, the TOS-BTO/PVDF composite displays I_20.3_/I_18.3_ ratios of 4 and 5.3 corresponding to 10% and 20% TOS-BTO nanoparticles, respectively. These results confirm that the surface modification process can lead to a significant proportion of the β-phase content.

The crystalline phase composition of the as-printed PVDF-based composites doped with various BTO and TOS-BTO nanoparticle weight fractions was further studied using FTIR spectroscopy, as shown in Figure 5c. The distinctive peaks at 614, 764, 855, and 976 cm^–1^ represent the α-phase, whereas the vibrational peak at 840 cm^–1^ represents the β-phase. The typical absorption strength of the α-phase in the printed PVDF-based composite was lower than that of pristine PVDF. Meanwhile, the absorption intensity of the β-phase is more apparent, demonstrating that the inclusion of BTO nanoparticles increased the crystal phase composition. Significantly, the TOS-BTO/PVDF nanocomposites exhibited lower β-phase peak intensities than the pristine BTO/PVDF nanocomposites. These results indicate that the incorporation of TOS-BTO nanoparticles into the PVDF polymer matrix may promote the formation of β-phase crystalline structures. In addition, the Beer–Lambert law was used to calculate the fraction of the electrically active β-phase (F(β)) in the as-printed nanocomposites:

(1) $$F\left(\beta \right)=\frac{A_{\beta }}{\left(\frac{K_{\beta }}{K_{\alpha }}\right)A_{\alpha }+A_{\beta }}$$

where A_α_ and A_β_ are the absorbance peaks at 762 and 840 cm^−1^, respectively, and K_α_ and K_β_ are 7.7 and 6.1 × 10^4^ cm^2^ mol^−1^, respectively. Figure 5d shows the F(β) values of the pristine PVDF and composite films with various BTO and TOS-BTO nanoparticle weight fractions. The F(β) value increased as the BTO content increased. The nanofillers in PVDF can aid the crystallization of the PVDF matrix by providing suitable nuclei in the polymeric chains. Nanocomposites with 20% TOS-BTO nanoparticles showed a considerable improvement in the F(β) value of 86.38%. Furthermore, the TOS-BTO/PVDF composite film displayed considerably higher F(β) values than its pristine BTO/PVDF counterpart, indicating that surface modification promoted β-phase crystalline formation in the PVDF matrix.

Figure 5e shows the phase transformation of the PVDF film with the addition of TOS-BTO nanoparticles. As illustrated schematically in Figure 5e, TOS was used to create an interfacial layer between the BTO nanoparticles and the PVDF polymer matrix. The hydroxyl groups in dopamine interacted with the BTO nanoparticles by creating covalent bonds, which produced an encapsulating layer surrounding the BTO surface by cross-linking, resulting in the low surface activity of the nanoparticles. During the mixing process, the positively charged methylene groups in the TOS molecules interacted with the negatively charged difluoromethylene groups in the PVDF chain to generate stable hydrogen bonds and form the electroactive β-phase, promoting compatibility and stability between the BTO nanoparticles and PVDF matrix and maintaining the alignment of the stabilized PVDF chains.

**Figure 6:**
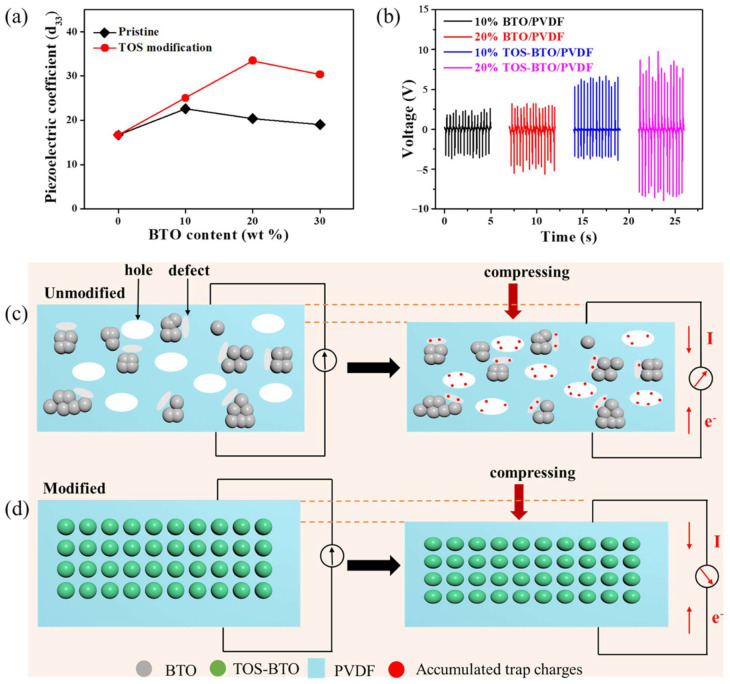
(**a**) Piezoelectric coefficient of PVDF-based composite films with various BTO or TOS-BTO nanoparticle contents. (**b**) Output voltages of PVDF-based composite films with various BTO or TOS-BTO nanoparticle contents. Schematic diagram of piezoelectric properties of the nanocomposite. (**c**) BTO/PVDF composite, (**d**) TOS-BTO/PVDF nanocomposite.

A quasistatic d_33_ meter was used to record the d_33_ of the screen-printed nanocomposites and investigate their piezoelectric capabilities, as shown in Figure 6a. The d_33_ value of the as-printed composites was significantly affected by the BTO nanoparticle concentration. In particular, the d_33_ value of the as-printed film increased from 16.7 pC/N for pure PVDF to 33.5 pC/N for the 20% TOS-BTO/PVDF as the TOS-BTO nanoparticle concentration increased. Additionally, compared to pristine BTO/PVDF, the d_33_ of the 20% TOS-BTO/PVDF-TrFE was 150% higher, demonstrating that adding TOS-BTO nanoparticles is another technique to enhance piezoelectric performance. The surface modification of nanoparticles significantly increases the piezoelectric properties of nanocomposites. The following are some of the factors that contributed to this improvement: BTO nanoparticles exhibit a higher d_33_ than PVDF. By combining the two materials, the piezoelectric capabilities of both phases are superior to that of pure PVDF. The high dielectric constant of the BTO nanoparticles, as ceramic particles, might focus the electric field onto the surrounding PVDF with a low dielectric constant. The stress concentration is another explanation for the increase in piezoelectricity. The addition of BTO nanoparticles increased the stiffness of the nanocomposites while decreasing the fracture strain, demonstrating the capacity of the nanocomposites to withstand high local stress. Furthermore, as shown in Figure 6c, numerous holes were caused by the loose contact between organic PVDF matrix and inorganic BTO nanoparticles, while there were many defects caused by the agglomerated BTO nanoparticles in the pristine BTO/PVDF nanocomposite. When the force was applied to the nanocomposite, the force-generated charges were accumulated and trapped in the holes and defects, resulting in the undesired piezoelectric properties. In contrast, as shown in Figure 6d, the TOS-BTO/PVDF nanocomposite achieved a homogenous dispersion of TOS-BTO by improving the compatibility between the nanofillers and polymer matrix following TOS coating on the BTO nanoparticle surface. Additionally, the TOS layer on the BTO surface created a stronger contact between the polymer matrix and nanofillers, resulting in fewer holes and defects in nanocomposites. Therefore, the decrease in the number of holes in the nanocomposites contributed to improved piezoelectricity by suppressing undesired cumulative trap charges. However, the d_33_ value reduced to 30.4 pC/N when the TOS-BTO nanoparticle concentration increased to 30 wt%. The explanation for the decline in d_33_ is that the nanoparticle aggregation has a negative electromechanical coupling impact. Furthermore, when a vertical pressing and releasing force of 50 N was applied at a frequency of 3 Hz, the open-circuit voltage (V_OC_) was measured, as shown in Figure 6b. As the number of TOS-BTO nanoparticles increased, the V_OC_ and d_33_ values also increased, indicating that the piezoelectric contribution to the electrical output was directly related to the d_33_ value. In particular, when the TOS-BTO nanoparticle concentration reached 20%, the highest peak-to-peak voltage of 20 V was achieved, which is almost two times higher than that of the pristine BTO/PVDF counterpart. Therefore, we selected the 20% TOS-BTO/PVDF PENG for subsequent experiments and compared it to the 20% BTO/PVDF PENG.

**Figure 7:**
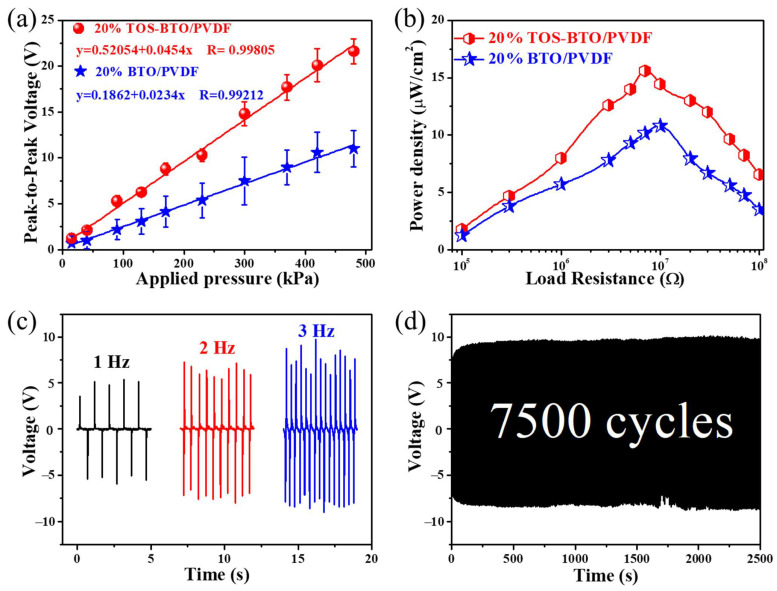
(**a**) Dependence of the output voltages of 20% TOS-BTO/PVDF PENGs and 20% BTO/PVDF PENGs on the external force. (**b**) Power density versus load resistance 10^5^–10^8^ Ω for 20% TOS-BTO/PVDF and 20% BTO/PVDF PENGs. (**c**) Frequency dependence of output voltages of 20% TOS-BTO/PVDF PENGs. (**d**) Output voltages of 20% TOS-BTO/PVDF PENGs for 7500 cycles under repeated impact force of a frequency of 3 Hz and a pressure of 500 kPa.

In order to study the device performance of the PVDF-based pressure sensors, the output piezoelectric signals were carefully measured under various stress conditions, as shown in Figure 7. Figure 7a depicts the voltage responses of the as-printed PENGs plotted as a function of the dynamic pressure ranging from 10 to 500 kPa at a frequency of 3 Hz. Clearly, the voltage output increases approximately linearly with the applied pressure across the entire range of measurements. In general, the piezoelectric potential and output voltage increase when the external impact force increases because of the larger deformation. The peak-to-peak voltages of the TOS-BTO/PVDF PENGs and pristine BTO/PVDF PENGs under dynamic compression stress of 500 kPa at 3 Hz were 20 and 9 V, respectively. The ultralarge linear response area of the as-printed sensor may offer a robust testing procedure and avoid complicated calibration methods. Furthermore, the slope of the linear graph may be used to calculate the voltage sensitivity of printed piezoelectric sensors: S = V/P, where V and P are the corresponding variances in the output voltage signal and applied pressure. According to the linear fitting result, the screen-printed TOS-BTO/PVDF PENGs exhibited better sensitivity (45.4 mV kPa^−1^) and voltage linearity (0.99805) than the pristine BTO/PVDF counterpart, demonstrating improved sensing capability compared to previously reported piezoelectric pressure sensors (Table 1). These results imply that the TOS surface-modification method may significantly increase the sensitivity of the printed device. In addition, as shown in Figure 7b, the output signals of the screen-printed PENGs were recorded with external load resistances ranging from 10 to 100 M under a 50 N load of 3 Hz to adequately analyze the power density of the screen-printed PENGs as an energy provider. The power density of the screen-printed PENGs can be calculated using the formula P = U^2^/(Rt), where P is the power density; U is the output voltage; R is the load resistance, and t is the effective area of the piezoelectric layer. There was an initial increase in power density, which subsequently decreased as the load resistance increased. At 5 M resistance, the highest power density of TOS-BTO/PVDF PENGs was 15.6 W/cm^−2^, which is ~150% higher than that of the pristine BTO/PVDF PENGs, confirming that the TOS surface-modification method can significantly enhance the energy harvesting capability. Furthermore, Figure 7c shows that as the impact frequency increased, the output voltages of the screen-printed TOS-BTO/PVDF PENGs increased. This phenomenon might have presumably been caused by the increasing frequency-induced strain rate of the nanocomposites. Moreover, to evaluate the durability of the screen-printed TOS-BTO/PVDF PENGs, the cycle test is carried out, and the result is shown in Figure 7d. After 7500 cycles of operation at a vertical pressing and releasing the pressure of 50 N at 3 Hz, the electric outputs exhibited no fluctuation or attenuation, demonstrating that the screen-printed TOS-BTO/PVDF PENGs display good mechanical robustness and endurance.

**Figure 8:**
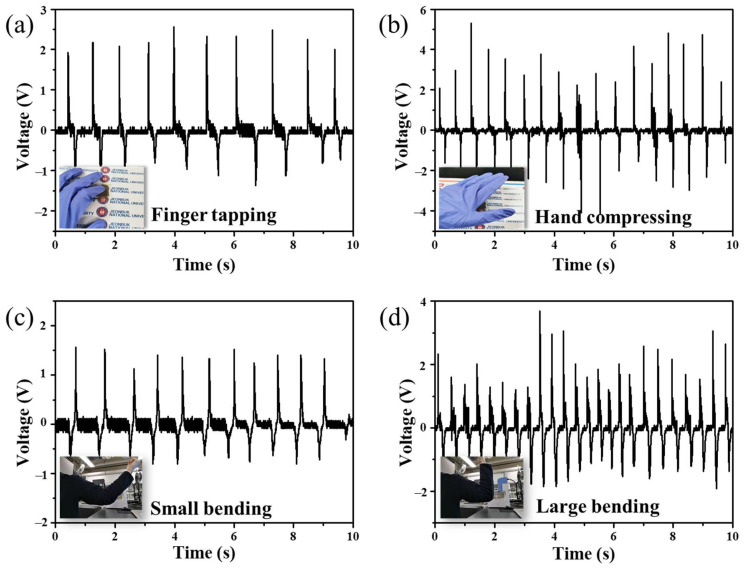
Output voltages generated by the screen-printed TOS-BTO/PVDF PENGs at (**a**) finger tapping and (**b**) hand compressing positions. Output voltages generated by the screen-printed TOS-BTO/PVDF PENGs at (**c**) small and (**d**) large bending positions.

The wearability and sensitivity of the screen-printed TOS-BTO/PVDF PENGs for active physiological monitoring were systematically investigated by measuring the electrical outputs under different human motions. The screen-printed TOS-BTO/PVDF PENGs created a piezoelectric output voltage under tapping and hand compression conditions, as shown in Figure 8a,b, respectively. Under the finger tapping and hand compression conditions, the screen-printed TOS-BTO/PVDF PENGs achieved output voltages of 2.6 and 4.5 V, respectively. As a result, the flexible printed sensor device described herein can gather biomechanical energy or detect human movement. The output voltages of the TOS-BTO/PVDF PENGs installed on a human arm are shown in Figure 8c,d under various arm bending conditions. Under small bending positions (bending angle of 30°), the printed TOS-BTO/PVDF PENGs generated output voltages of 1.5 V, while the printed device generated output voltage of 2.6 V under large bending position (bending angle of 90°), respectively. During massive bending, the TOS-BTO nanoparticles and PVDF polymer were subjected to much more strain, resulting in a larger electrical output. Therefore, this flexible screen-printed TOS-BTO/PVDF PENG is a viable option for real-time/practical applications, such as harvesting biomechanical energy or detecting human body movements.

## 4. Conclusions

A flexible and high-performance nanocomposite PENG based on a TOS-BTO/PVDF composite film was fabricated via screen printing. An organic TOS functional group in the polymer matrix assists in the uniform dispersion of nanoinorganic fillers. The degree of charge annihilation that occurs during the charge transfer from inorganic particles to the polymer matrix is reduced in the composites. As a result, the TOS-modified BTO/PVDF pressure sensor exhibited a noticeable increase in the output voltage (20 V) and output power density (15.6 μW cm^−2^), which were considerably higher than those of the pristine BTO/PVDF counterpart. These findings demonstrate the importance of the surface modification of inorganic nanofillers implanted in PENGs. These gadgets may also be used to actively detect human movements because of their high flexibility and sensitivity when manufactured on demand. These screen-printed PENGs demonstrate potential as future touch sensors manufactured using additive manufacturing processes in printed electronics applications.

## Figures and Tables

**Table 1:** Comparison of the output performance of PVDF-based PENGs.

Method	Materials	d_33_ (pC/N)	Output Voltage	Sensitivity	Ref.
Electrospinning	PDA@BTO/PVDF-TrFE	N/A	6 V; 700 N	N/A	[38]
Casting	PDA@BTO/PVDF	N/A	24 V; 234 N	N/A	[39]
Casting	BTO@C/PVDF-TrFE	30	17 V; bending	N/A	[42]
Casting	Li-ZnO/PVDF	N/A	3.4 V; hand	N/A	[43]
Electrospinning	PMMA@BTO/PVDF	20	12.6 V; bending	N/A	[44]
printing	TPPC/PVDF	1.85	6.62 V; 5 m/s^2^	N/A	[45]
printing	IL/PVDF	N/A	4.2 V; hand	N/A	[46]
printing	BTO/PVDF-TrFE	N/A	13.2 V; 50 N	26.4 mV/kPa	[47]
printing	TOS-BTO/PVDF	33.5	20 V; 50 N	45.4 mV/kPa	This study

## Data Availability

The data are available upon request from the corresponding authors.

## References

[1] Khan Y., Thielens A., Muin S., Ting J., Baumbauer C., Arias A.C. (2020). A New Frontier of Printed Electronics: Flexible Hybrid Electronics. Adv. Mater..

[2] He P., Cao J., Ding H., Liu C., Neilson J., Li Z., Kinloch I.A., Derby B. (2019). Screen-Printing of a Highly Conductive Graphene Ink for Flexible Printed Electronics. ACS Appl. Mater. Interfaces.

[3] Goh G.L., Saengchairat N., Agarwala S., Yeong W.Y., Tran T. (2019). Sessile droplets containing carbon nanotubes: A study of evaporation dynamics and CNT alignment for printed electronics. Nanoscale.

[4] Liang X., Li H., Dou J., Wang Q., He W., Wang C., Li D., Lin J.M., Zhang Y. (2020). Stable and Biocompatible Carbon Nanotube Ink Mediated by Silk Protein for Printed Electronics. Adv. Mater..

[5] Kuang X., Roach D.J., Wu J., Hamel C.M., Ding Z., Wang T., Dunn M.L., Qi H.J. (2019). Advances in 4D Printing: Materials and Applications. Adv. Funct. Mater..

[6] Zolfagharian A., Kaynak A., Kouzani A. (2020). Closed-loop 4D-printed soft robots. Mater. Des..

[7] Ryan K.R., Down M.P., Banks C.E. (2021). Future of additive manufacturing: Overview of 4D and 3D printed smart and advanced materials and their applications. Chem. Eng. J..

[8] Zhou Y., Parker C.B., Joshi P., Naskar A.K., Glass J.T., Cao C. (2021). 4D Printing of Stretchable Supercapacitors via Hybrid Composite Materials. Adv. Mater. Technol..

[9] Tang X., Murali G., Lee H., Park S., Lee S., Oh S.M., Lee J., Ko T.Y., Koo C.M., Jeong Y.J. (2021). Engineering Aggregation-Resistant MXene Nanosheets As Highly Conductive and Stable Inks for All-Printed Electronics. Adv. Funct. Mater..

[10] Carey T., Arbab A., Anzi L., Bristow H., Hui F., Bohm S., Wyatt-Moon G., Flewitt A., Wadsworth A., Gasparini N. (2021). Inkjet Printed Circuits with 2D Semiconductor Inks for High-Performance Electronics. Adv. Electron. Mater..

[11] Heikkinen I.T.S., Kauppinen C., Liu Z., Asikainen S.M., Spoljaric S., Seppälä J.V., Savin H., Pearce J.M. (2018). Chemical compatibility of fused filament fabrication-based 3-D printed components with solutions commonly used in semiconductor wet processing. Addit. Manuf..

[12] Carlos E., Leppäniemi J., Sneck A., Alastalo A., Deuermeier J., Branquinho R., Martins R., Fortunato E. (2020). Printed, Highly Stable Metal Oxide Thin-Film Transistors with Ultra-Thin High-κ Oxide Dielectric. Adv. Electron. Mater..

[13] Schmidt G.C., Panicker P.M., Qiu X., Benjamin A.J., Quintana Soler R.A., Wils I., Hübler A.C. (2021). Paper-Embedded Roll-to-Roll Mass Printed Piezoelectric Transducers. Adv. Mater..

[14] Aliqué M., Simão C.D., Murillo G., Moya A. (2021). Fully-Printed Piezoelectric Devices for Flexible Electronics Applications. Adv. Mater. Technol..

[15] Chen Y., Xie B., Long J., Kuang Y., Chen X., Hou M., Gao J., Zhou S., Fan B., He Y. (2021). Interfacial Laser-Induced Graphene Enabling High-Performance Liquid-Solid Triboelectric Nanogenerator. Adv. Mater..

[16] Liu Z., Li J., Liu X. (2020). Novel Functionalized BN Nanosheets/Epoxy Composites with Advanced Thermal Conductivity and Mechanical Properties. ACS Appl. Mater. Interfaces.

[17] Li H., Song H., Long M., Saeed G., Lim S. (2021). Mortise-tenon joint structured hydrophobic surface-functionalized barium titanate/polyvinylidene fluoride nanocomposites for printed self-powered wearable sensors. Nanoscale.

[18] Li H., Lim S. (2021). Boosting performance of self-polarized fully printed piezoelectric nanogenerators via modulated strength of hydrogen bonding interactions. Nanomaterials.

[19] Gao X., Yang J., Wu J., Xin X., Li Z., Yuan X., Shen X., Dong S. (2020). Piezoelectric Actuators and Motors: Materials, Designs, and Applications. Adv. Mater. Technol..

[20] Lu L., Ding W., Liu J., Yang B. (2020). Flexible PVDF based piezoelectric nanogenerators. Nano Energy.

[21] Cho Y., Jeong J., Choi M., Baek G., Park S., Choi H., Ahn S., Cha S., Kim T., Kang D.S. (2022). BaTiO3@PVDF-TrFE nanocomposites with efficient orientation prepared via phase separation nano-coating method for piezoelectric performance improvement and application to 3D-PENG. Chem. Eng. J..

[22] Wang R., Xie X., Xu C., Lin Y., You D., Chen J., Li Z., Shi Z., Cui Q., Wang M. (2022). Bi-piezoelectric effect assisted ZnO nanorods/PVDF-HFP spongy photocatalyst for enhanced performance on degrading organic pollutant. Chem. Eng. J..

[23] Marandi M., Tarbutton J. (2019). Additive manufacturing of single- And double-layer piezoelectric PVDF-TrFE copolymer sensors. Procedia Manuf..

[24] Song L., Dai R., Li Y., Wang Q., Zhang C. (2021). Polyvinylidene Fluoride Energy Harvester with Boosting Piezoelectric Performance through 3D Printed Biomimetic Bone Structures. ACS Sustain. Chem. Eng..

[25] Martins P., Lopes A.C., Lanceros-Mendez S. (2014). Electroactive phases of poly(vinylidene fluoride): Determination, processing and applications. Prog. Polym. Sci..

[26] Pi Z., Zhang J., Wen C., Zhang Z., Wu D. (2014). Flexible piezoelectric nanogenerator made of poly(vinylidenefluoride-co-trifluoroethylene) (PVDF-TrFE) thin film. Nano Energy.

[27] Ali M., Prakash D., Zillger T., Singh P.K., Hübler A.C. (2014). Printed Piezoelectric Energy Harvesting Device. Adv. Energy Mater..

[28] Gee S., Johnson B., Smith A.L. (2018). Optimizing electrospinning parameters for piezoelectric PVDF nanofiber membranes. J. Memb. Sci..

[29] Jin L., Ma S., Deng W., Yan C., Yang T., Chu X., Tian G., Xiong D., Lu J., Yang W. (2018). Polarization-free high-crystallization β-PVDF piezoelectric nanogenerator toward self-powered 3D acceleration sensor. Nano Energy.

[30] Khan F., Kowalchik T., Roundy S., Warren R. (2021). Stretching-induced phase transitions in barium titanate-poly(vinylidene fluoride) flexible composite piezoelectric films. Scr. Mater..

[31] Kitsara M., Blanquer A., Murillo G., Humblot V., De Bragança Vieira S., Nogués C., Ibáñez E., Esteve J., Barrios L. (2019). Permanently hydrophilic, piezoelectric PVDF nanofibrous scaffolds promoting unaided electromechanical stimulation on osteoblasts. Nanoscale.

[32] Choi M.H., Yang S.C. (2018). CoFe2O4 nanofiller effect on β-phase formation of PVDF matrix for polymer-based magnetoelectric composites. Mater. Lett..

[33] Cai J., Hu N., Wu L., Liu Y., Li Y., Ning H., Liu X., Lin L. (2019). Preparing carbon black/graphene/PVDF-HFP hybrid composite films of high piezoelectricity for energy harvesting technology. Compos. Part A Appl. Sci. Manuf..

[34] Chen J.X., Li J.W., Cheng C.C., Chiu C.W. (2022). Piezoelectric Property Enhancement of PZT/Poly(vinylidenefluoride-co-trifluoroethylene) Hybrid Films for Flexible Piezoelectric Energy Harvesters. ACS Omega.

[35] Song S., Li Y., Wang Q., Zhang C. (2021). Boosting piezoelectric performance with a new selective laser sintering 3D printable PVDF/graphene nanocomposite. Compos. Part A Appl. Sci. Manuf..

[36] Zhang J.H., Zhou Z., Li J., Shen B., Zhu T., Gao X., Tao R., Guo X., Hu X., Shi Y. (2022). Coupling Enhanced Performance of Triboelectric-Piezoelectric Hybrid Nanogenerator Based on Nanoporous Film of Poly(vinylidene fluoride)/BaTiO3Composite Electrospun Fibers. ACS Mater. Lett..

[37] Hu X., Yan X., Gong L., Wang F., Xu Y., Feng L., Zhang D., Jiang Y. (2019). Improved Piezoelectric Sensing Performance of P(VDF-TrFE) Nanofibers by Utilizing BTO Nanoparticles and Penetrated Electrodes. ACS Appl. Mater. Interfaces.

[38] Guan X., Xu B., Gong J. (2020). Hierarchically architected polydopamine modified BaTiO3@P(VDF-TrFE) nanocomposite fiber mats for flexible piezoelectric nanogenerators and self-powered sensors. Nano Energy.

[39] Yang Y., Pan H., Xie G., Jiang Y., Chen C., Su Y., Wang Y., Tai H. (2020). Flexible piezoelectric pressure sensor based on polydopamine-modified BaTiO3/PVDF composite film for human motion monitoring. Sens. Actuators A Phys..

[40] Du D., Tang Y., Yang L., Tang C. (2020). Effects of different grafting density of amino silane coupling agents on thermomechanical properties of cross-linked epoxy resin. Polymers.

[41] Nuryono N., Qomariyah A., Kim W., Otomo R., Rusdiarso B., Kamiya Y. (2019). Octyl and propylsulfonic acid co-fixed Fe3O4@SiO2 as a magnetically separable, highly active and reusable solid acid catalyst in water. Mol. Catal..

[42] Zhou Z., Zhang Z., Zhang Q., Yang H., Zhu Y., Wang Y., Chen L. (2020). Controllable Core-Shell BaTiO3@Carbon Nanoparticle-Enabled P(VDF-TrFE) Composites: A Cost-Effective Approach to High-Performance Piezoelectric Nanogenerators. ACS Appl. Mater. Interfaces.

[43] Jin C., Hao N., Xu Z., Trase I., Nie Y., Dong L., Closson A., Chen Z., Zhang J.X.J. (2020). Flexible piezoelectric nanogenerators using metal-doped ZnO-PVDF films. Sens. Actuators A Phys..

[44] Shi K., Chai B., Zou H., Shen P., Sun B., Jiang P., Shi Z., Huang X. (2021). Interface induced performance enhancement in flexible BaTiO3/PVDF-TrFE based piezoelectric nanogenerators. Nano Energy.

[45] Pei H., Xie Y., Xiong Y., Lv Q., Chen Y. (2021). A novel polarization-free 3D printing strategy for fabrication of poly (Vinylidene fluoride) based nanocomposite piezoelectric energy harvester. Compos. Part B Eng..

[46] Liu X., Shang Y., Zhang J., Zhang C. (2021). Ionic Liquid-Assisted 3D Printing of Self-Polarized β-PVDF for Flexible Piezoelectric Energy Harvesting. ACS Appl. Mater. Interfaces.

[47] Zhou X., Parida K., Halevi O., Liu Y., Xiong J., Magdassi S., Lee P.S. (2020). All 3D-printed stretchable piezoelectric nanogenerator with non-protruding kirigami structure. Nano Energy.

